# Identification of Glial Activation Markers by Comparison of Transcriptome Changes between Astrocytes and Microglia following Innate Immune Stimulation

**DOI:** 10.1371/journal.pone.0127336

**Published:** 2015-07-27

**Authors:** Silvia Madeddu, Tyson A. Woods, Piyali Mukherjee, Dan Sturdevant, Niranjan B. Butchi, Karin E. Peterson

**Affiliations:** 1 Laboratory of Persistent Viral Diseases, Rocky Mountain Laboratories, National Institute of Allergy and Infectious Diseases (NIAID), National Institutes of Health (NIH), Hamilton, Montana, United States of America; 2 Research Technologies Branch, RML, NIAID, NIH, Hamilton, Montana, United States of America; National Brain Research Center, INDIA

## Abstract

The activation of astrocytes and microglia is often associated with diseases of the central nervous system (CNS). Understanding how activation alters the transcriptome of these cells may offer valuable insight regarding how activation of these cells mediate neurological damage. Furthermore, identifying common and unique pathways of gene expression during activation may provide new insight into the distinct roles these cells have in the CNS during infection and inflammation. Since recent studies indicate that TLR7 recognizes not only viral RNA but also microRNAs that are released by damaged neurons and elevated during neurological diseases, we first examined the response of glial cells to TLR7 stimulation using microarray analysis. Microglia were found to generate a much stronger response to TLR7 activation than astrocytes, both in the number of genes induced as well as fold induction. Although the primary pathways induced by both cell types were directly linked to immune responses, microglia also induced pathways associated with cellular proliferation, while astrocytes did not. Targeted analysis of a subset of the upregulated genes identified unique mRNA, including *Ifi202b* which was only upregulated by microglia and was found to be induced during both retroviral and bunyavirus infections in the CNS. In addition, other genes including *Birc3* and *Gpr84* as well as two expressed sequences AW112010 and BC023105 were found to be induced in both microglia and astrocytes and were upregulated in the CNS following virus infection. Thus, expression of these genes may a useful measurement of glial activation during insult or injury to the CNS.

## Introduction

Neuroinflammation, including the activation of microglia and astrocytes and the production of proinflammatory cytokines, is commonly found in association with infection or disease in the central nervous system (CNS) [1–5]. The initiation of these neuroinflammatory responses are often mediated by pattern recognition receptors (PRRs), such as membrane bound toll-like receptors (TLRs) as well as cytoplasmic RNA and DNA sensors [1, 6, 7]. These PRRs are stimulated during infections of the CNS by pathogen associated molecular patterns (PAMPs); structural motifs in nucleic acids, lipids or proteins from pathogens that are not commonly found in a eukaryotic cell [8–10]. Damage-associated molecular patterns (DAMPs), such as nucleic acids from apoptotic cells or secreted micro-RNAs, have also been associated with neurological disease or damage and can also stimulate PRRs, particularly endosomal toll-like receptor 7 (TLR7) and TLR9 [11–13]. Examining how stimulation of these receptors mediates neuroinflammatory responses is important in determining the mechanisms of pathogenesis for diseases of the CNS.

The CNS has limited interactions with peripheral immune cells due to the lack of lymphatic vessels and the presence of blood–brain and blood–cerebrospinal fluid (CSF) barriers that limit the influx of cells and protein to the CNS. Instead, cells intrinsic to the brain such as microglia and astrocytes are often the primary responders to infection or damage in the CNS. Activated astrocytes and microglia are both found in a number of neurological disorders and their activation state often correlates with the severity of disease [1–5]. Furthermore, both of these cell types have important roles in inducing neuroinflammation and regulating neuropathogenesis [1, 4, 5, 14].

Microglia and astrocytes are distinct in their cellular origins and functions in the CNS. Microglia are derived early during development from immature progenitors in the yolk sac and have an important role in synaptic pruning of neurons in the developing brain [15]. These cells then populate the CNS and persist for the entire life of the organism with only limited turnover from bone-marrow derived monocytes. In the mature CNS, microglia have a ramified morphology and only become amoeboid in shape upon activation. They actively respond to infection or damage in the CNS, phagocytizing microorganisms, dying cells and cellular debris as well as producing inflammatory mediators [8, 9, 15]. In contrast to microglia, astrocytes are neuroectodermal in origin and are responsible for a wide variety of functions in the CNS [2]. For example, they regulate transendothelial cell migration across the blood-brain barrier (BBB) and contribute to regulation of synaptic activity throughout the brain. Upon activation, astrocytes upregulate expression of glial fibrillary acidic protein (GFAP) and undergo process extension and interdigitation. Excessive activation of astrocytes during infection or inflammation can result in astrocyte scarring, leading to long term tissue damage [2, 10, 16–18].

The differential activation of microglia and astrocytes may indicate unique roles in their ability to detect and respond to pathogen infection of the CNS. Microarray analysis of unstimulated microglia and astrocytes demonstrated unique transcriptomes of unstimulated cells, with microglia associating more closely with bone marrow monocytes and dendritic cells compared to astrocytes or neurons [19]. However, both microglia and astrocytes are capable of recognizing multiple viral and bacterial infections [10, 20, 21] and secrete high levels of cytokines following activation [5, 20, 22]. Indeed, our previous studies showed similar induction of cytokines following TLR stimulation of astrocytes or microglia [20]. However, a comprehensive analysis of the similarities and differences in the microglial and astrocytic responses to innate immune stimulation would provide a better understanding of the role of these cells in responding to pathogenic insults in the CNS.

To better differentiate the response of microglia and astrocytes to immune stimulation, we analyzed the transcriptome of both cell types using microarrays following innate immune activation. We focused primarily on TLR7 stimulation due to the role of TLR7 in recognizing viral and bacterial RNAs as well as cellular microRNAs (miRNAs) [23–27]. These cellular miRNAs may serve as DAMPs to induce innate immune responses in the CNS, and recent studies have found increased expression of certain miRNAs, such as *let-7b*, in the CSF of Alzheimer’s patients as well as the release of *let-7b* from dying neurons [13, 28]. Thus, TLR7 may be involved in mediating immune responses to both infectious and non-infectious diseases of the CNS.

In our current study, we found that TLR7 activation induced a more robust response in microglia compared to astrocytes with the significant upregulation or down-regulation of a larger number of genes and a higher ratio of activation compared to mock controls. Real-time PCR analysis confirmed the higher ratio of gene expression by microglia, although it did detect gene expression in astrocytes that was not detectable by microarray. Comparison of TLR7 to another endosomal TLR, TLR9, indicated that the increased response by microglia was consistent for both receptors. Analysis of several of the upregulated genes in both cell types revealed potential markers for both microglia and astrocyte activation during viral activation. The findings from these studies provide a better understanding in how TLR activation alters the function of both astrocytes and microglia as well as identifying genes that may be potential markers for glial activation in vivo and in vitro.

## Materials and Methods

### Ethics statement

All animal research was carried out in adherence with protocols approved by the National Institutes of Health Rocky Mountain Laboratories Animal Care and Use Committee, animal protocols 2012–47 and 2012–46. The method of euthanasia for neonatal mice used for generation of primary glial cell cultures was hypothermia, followed by decapitation following the NIH guidelines since neonatal mice are not sensitive to inhalant anesthetics.

### Virus infection of mice

La Crosse Virus (LACV) human 1978 stock was a kind gift of Richard Bennett (NIAID, NIH) and has been previously described [29, 30]. Mice were infected with 10^3^ plaque forming units (PFU) of LACV in PBS at 21 days of age by intraperitoneal (200 μl/mouse) injection. At the onset of neurological disease (6–10 days post infection), brain tissue was removed, frozen in liquid nitrogen and processed for RNA. Age-matched controls were inoculated with lysates from uninfected Vero cells. For retrovirus infection, Inbred Rocky Mountain White (IRW) mice were infected with 10^4^ focus forming units (FFU) of the neurovirulent Friend virus, BE [31] within 48 hours of birth. Tissues were removed at the time of onset of neurological disease onset (18–24 days post infection). Age-matched controls were inoculated with supernatants from uninfected Mus dunni cells. Tissues were frozen in liquid nitrogen prior to processing for RNA analysis.

### Isolation and Culturing of Cortical Astrocytes and Microglia

Astrocyte and microglia cultures were prepared from the brain cortex of 1–2 day old Inbred Rocky Mountain White (IRW) mice as previously described [20]. In brief, brain tissue was removed from multiple animals at 2 days of age and placed in ice cold phosphate buffered saline (PBS). Hind brains, mid brains, and meninges were dissected out. Cerebral cortices were transferred to a 15-mL conical tube containing 2% glucose in PBS and made into a single cell suspension. Cells were pelleted by centrifugation at 500 g-force for 5 min. Cells from two brain cortices were suspended in 2 mL of 70% percoll and transferred to the bottom of a 0–30% Percoll step gradient. The gradients were centrifuged at 500g-force for 20 min. Cells between the 0% and 30% Percoll layers were rich in astrocytes and were seeded at 2 x 10^5^ cells per Primaria T-25 flasks (BD Bioscience). The microglia cell populations collected between 30% and 70% percoll layers were seeded at 5 x 10^5^ cells per Primaria T-25 flasks. When cells became confluent after 7–10 days of culture, flasks containing astrocyte rich cells (0/30 fraction) were orbitally shaken overnight at 250 rpm to remove any remaining contaminating microglia or oligodendrocytes. Astrocytes were then treated with 0.25% Trypsin-EDTA (Gibco), reseeded in 12-well Cell-bind plates (Corning). Microglia were removed from confluent T-25 flasks using a cell scraper and reseeded in 12-well cell bind plates. The purity of astrocyte and microglia cultures were confirmed by intracellular flow cytometry, and were consistently greater than 93% GFAP positive or 95% F4/80 positive, respectively (data not shown).

### TLR Agonists

The TLR7 agonist imiquimod (R837) and TLR9 agonist type B CpG-ODN 1826 [5’-TCC ATG ACG TTC CTG ACG TT-3’] were purchased from InvivoGen. All the agonists were suspended in endotoxin-free water, aliquoted, and stored at -20°C. Just before use, agonists were diluted in media specific for either astrocytes or microglia.

### Culture and stimulation of astrocyte and microglia cultures

Astrocyte cultures were maintained in Dulbecco’s modified Eagle’s medium (Sigma-Aldrich) containing 4,500 mg glucose/L, 110 mg sodium pyruvate/L, 0.584 g L-glutamine/L, supplemented with 10% inactivated fetal bovine serum (Atlas Biologicals) and 1% penicillin-streptomycin (Gibco). Microglia-specific media contained 20% LADMAC culture supernatant (mouse bone marrow cells producing macrophage colony stimulating factor/M-CSF) in addition to the media used for astrocyte cultures [20]. Astrocytes and microglia were treated with either 5 μM imiquimod or 80 nM of CpG-ODN 1826. Cells were lysed at 6 hrs post stimulation for RNA analysis.

### Microarray analysis

Individual wells from six-well plates of microglia or astrocytes prepared from multiple mice were randomly assigned to either mock or imiquimod stimulation groups. Six replicates per group were used for microarray analysis. Microarray analysis was performed by the Genomics Unit of the Research Technologies branch, NIAID using GeneChip Mouse Gene 1.0 ST Array from Affymetrix. The raw data set was analyzed using Partek Genomics Suite (Partek Inc., St. Louis, MO), in which the raw data was quantile normalized to remove non-biological variation [32] and an ANOVA was run for the comparisons of interest. These results were multiple test corrected (significance level 0.05) using the Benjamini and Hochberg false discovery rate step-up method for producing the corrected p-value cutoff points [33]. All genes upregulated or down-regulated greater than 2 fold shown in Tables 1–3 had significant (P<0.05) differences between mock and imiquimod stimulated samples. Data were analyzed through the use of QIAGEN’s Ingenuity Pathway Analysis (IPA) (Qiagen) to identify and compare pathways that were altered by TLR7 stimulation of astrocytes or microglia. Z-scores, “which determine whether upstream transcription regulators of a pathway have significantly more activated predictions than they have inhibited predictions were considered significant if greater than 2 or less than -2. These scores were used to define pathways activated by TLR7 stimulation of microglia or astrocytes.

**Table 1:** Ingenuity Pathway Analysis of disease and biomarkers pathways induced by TLR7 stimulation.

	Activation Z score^a^													
Pathway	microglia	astrocyte	average	difference^b^	Top genes in each pathway:									
**Highest Z scores pathways**					Upregulated					Down-regulated				
**microglia:**														
activation of blood cells	**6.04**	5.62	5.83	0.42	*Il6*	*Il1b*	*Il1a*	*Il12b*	*Ptgs2*	*Cd300lf*	*Cd28*	*Cd180*	*Gmnn*	*Cx3cr1*
activation of leukocytes	**5.88**	5.71	5.80	0.18	*Il6*	*Il1b*	*Il1a*	*Il12b*	*Ptgs2*	*Havcr2*	*Cd300lf*	*CD28*	*Cd180*	
activation of phagocytes	**5.33**	4.71	5.02	0.63	*Il6*	*Il1b*	*Il1a*	*Ptgs2*	*Cxcl1*	*Havcr2*	*Cd300lf*			
recruitment of cells	**4.65**	4.13	4.39	0.52	*Il6*	*Il1b*	*Il1a*	*Cd69*	*Ptgs2*	*S1pr1*	*Cd28*	*Cxcr4*	*Cx3cr1*	*Gcnt1*
migration of cells	**4.60**	3.92	4.26	0.68	*Il6*	*Il1b*	*Il1a*	*Il12b*	*Cd69*	*Hpgd*	*Ccnd1*	*Cx3cr1*	*Plau*	*Gcnt1*
**astrocytes**														
activation of leukocytes	5.88	**5.71**	5.80	0.18	*Il1a*	*Cxcl3*	*Fpr1*	*Il1b*	*Clec4e*	*Havcr2*				
activation of blood cells	6.04	**5.62**	5.83	0.42	*Il1a*	*Cxcl3*	*Fpr1*	*Il1b*	*Clec4e*	*Cx3cr1*	*Havcr2*			
activation of phagocytes	5.33	**4.71**	5.02	0.63	*Il1a*	*Cxcl3*	*Fpr1*	*Il1b*	*Clec4e*	*Havcr2*				
cell movement of monocytes	4.16	**4.16**	4.16	0.00	*Cxcl3*	*Ccl3l1*	*Fpr1*	*Il1b*	*Cxcl10*	*—*				
differentiation of cells	3.68	**4.14**	3.91	-0.47	*Il1a*	*Cd69*	*Ccl3l1*	*Il1b*	*Cxcl10*	*Havcr2*	*Gcnt1*			
**Greatest differences in Z scores**														
**microglia**														
lymphatic node tumor	2.41	-0.29	1.06	**2.71**	*Il6*	*Cd69*	*Tnf*	*Cd40*	*Gadd45b*	*Msh2*	*Cxcr4*	*Pola1*	*Klhl6*	*Ccnd1*
non-Hodgkin's disease	2.41	-0.29	1.06	**2.71**	*Il6*	*Cd69*	*Tnf*	*Gadd45b*	*Cxcl10*	*Msh2*	*Cxcr4*	*Pola1*	*Klhl6*	*Ccnd1*
lymphomagenesis	1.91	0.01	0.96	**1.90**	*Il6*	*Cd69*	*Nos2*	*Tnf*	*Cd40*	*Msh2*	*Cxcr4*	*Pola1*	*Klhl6*	*Ccnd1*
malignant lymphocytic neoplasm	1.91	0.01	0.96	**1.90**	*Il6*	*Il1b*	*Il1a*	*Il12b*	*Cd69*	*Adrb2*	*Ccnd1*	*Cx3cr1*	*Plau*	*Gcnt1*
**astrocytes**														
quantity of IgG3	0.13	1.63	0.88	**-1.51**	*Tnf*	*Tlr2*	*Ptgs2*	*Pik3ap1*	*Rel*	—				
cell cycle progression	0.36	1.96	1.16	**-1.60**	*Il1a*	*Il1b*	*Cxcl10*	*Tnf*	*Il6*	*Hpgd*				
quantity of lymphatic system cells	0.83	2.46	1.65	**-1.62**	*Il1a*	*Tnf*	*Il6*	*Nfkbia*	*Pik3ap1*	—				
quantity of interleukin	0.50	1.65	1.07	**-1.14**	*Il1b*	*Ccl2*	*Tnf*	*Il6*	*Tlr2*	—				

a: Z score for each data set as calculated by IPA software. A score above 2.00 is significant. Bold scores for each group were used for ranking

b: difference in Z scores calculated by subtracting astrocyte Z score from microglia Z score

**Table 2:** Comparison of fold change in mRNA levels between microarray and RT- PCR^a^.

			Microarray			real-time PCR^f^		
	Rank^b^	Gene	micro^c^	astro^d^	Difference^e^	micro	astro	Difference
Fig 2(both)	4	***Cd69***	**22.1**	10.4	**2.1**	**37.7**	**205.0**	*0*.*18*
	6	***AW112010***	**20.7**	2.5	**8.3**	**106.2**	**55.5**	**1.91**
	8	***Ptgs2 (Cox 2)***	**19.1**	2.4	**7.9**	**30.6**	**4.4**	**6.95**
	9	***Irg1***	**15.9**	5.2	**3.1**	**193.9**	**63.2**	**3.07**
	13	***Fpr1***	**9.4**	6.1	1.5	**79.2**	**40.2**	1.97
	14	***Gbp5***	**10.5**	4.2	**2.5**	**443.3**	**28.2**	**15.74**
	22	***Tnfrsf1b***	**5.5**	3.0	1.8	**4.7**	**10.3**	*0*.*46*
	23	***Irak3***	**5.3**	3.0	1.7	**7.1**	**16.0**	*0*.*44*
	25	***Malt1***	**5.5**	3.1	1.8	**11.4**	**8.5**	1.34
	27	***Gpr84***	**4.4**	3.1	1.4	**21.2**	**14.9**	1.43
	29	***Nfkbiz***	**4.5**	2.4	1.9	**28.2**	**14.2**	1.98
	32	***Tnfaip2***	**2.9**	3.3	0.9	**2.9**	**13.6**	*0*.*22*
	35	***Casp4***	**3.7**	2.2	1.7	**7.3**	**4.7**	1.55
	36	***Birc3***	**3.5**	2.2	1.6	**4.4**	**6.4**	0.69
Fig 3 (microglia)	1	***Marco***	**17.2**	1.3	**13.0**	**562.0**	**68.9**	**8.16**
	4	***Saa3***	**13.0**	1.1	**11.8**	**3,075.7**	**186.9**	**16.46**
	6	***Gbp2***	**10.0**	1.9	**5.4**	**31.7**	**8.2**	**3.88**
	9	***Traf1***	**9.0**	1.9	**4.8**	**158.8**	**6.8**	**23.29**
	30	***Nlrp3***	**3.9**	1.9	**2.1**	**7.4**	**19.9**	*0*.*37*
	31	***Ifit1***	**3.8**	1.3	**2.9**	**5.0**	**5.6**	0.88
	50	***Ifi202b***	**3.3**	1.1	**2.9**	**4.2**	1.8	**2.35**
	69	***Zc3h12c***	**2.9**	1.7	**1.7**	**5.0**	**6.7**	0.75
Fig 4(astrocytes)	3	***Rapgef5***	1.1	**2.5**	*0*.*4*	0.9	**3.1**	*0*.*29*
	4	***BC023105***	1.1	**2.2**	0.5	**31.9**	**6.6**	**4.84**
	7	***Elovl7***	1.5	**2.1**	0.7	1.9	**4.4**	*0*.*43*
	8	***Cxcl11***	1.3	**2.1**	0.6	**61.7**	**20.2**	**3.06**

a: bold cells: increase >2 fold, italicize cells: decrease > 2 fold

b: rank of genes in Figs 2, 3 and 4

c: fold change from mock stimulated microglia as determined by microarray

d: fold change from mock stimulated astrocytes as determined by microarray

e: difference between microglia and astrocytes in gene expression

f: fold change in TLR7-stimulated microglia or astrocytes compared to mock stimulated cells using real-time PCR

**Table 3:** mRNA expression following TLR9 ligand stimulation^a^.

			CpG-ODN (fold)^b^			Imiquimod/CpG-ODN^d^	
	Rank	Gene	micro	astro	Difference^c^	microglia	astrocytes
Fig 2 (both)	4	***Cd69***	**126.1**	**89.9**	1.4	*0*.*30*	**2.28**
	6	***AW112010***	**110.3**	**14.3**	**7.7**	0.96	**3.89**
	8	***Ptgs2 (Cox 2)***	**18.8**	**2.2**	**8.7**	1.62	**2.03**
	9	***Irg1***	**220.8**	**27.7**	**8.0**	0.88	**2.28**
	13	***Fpr1***	**98.5**	**22.0**	**4.5**	0.80	1.83
	14	***Gbp5***	**268.8**	**10.8**	**24.9**	1.65	**2.61**
	22	***Tnfrsf1b***	**3.9**	**5.2**	0.7	1.22	1.99
	23	***Irak3***	**4.9**	**7.2**	0.7	1.42	**2.22**
	25	***Malt1***	**7.1**	**3.0**	**2.3**	1.61	**2.79**
	27	***Gpr84***	**27.0**	**8.1**	**3.3**	0.79	1.84
	29	***Nfkbiz***	**39.6**	**9.0**	**4.4**	0.71	1.58
	32	***Tnfaip2***	**5.9**	**8.9**	0.7	0.50	1.53
	35	***Casp4***	**7.3**	**3.0**	**2.4**	1.01	1.60
	36	***Birc3***	**5.8**	**4.4**	1.3	0.76	1.47
Fig 3 (microglia)	1	***Marco***	**620.0**	**26.3**	**23.6**	0.91	**2.62**
	4	***Saa3***	**692.9**	**45.8**	**15.1**	**4.44**	**4.08**
	6	***Gbp2***	**25.5**	**3.8**	**6.7**	1.24	**2.13**
	9	***Traf1***	**74.8**	**2.7**	**27.4**	**2.12**	**2.50**
	30	***Nlrp3***	**15.4**	**10.0**	1.6	*0*.*48*	1.99
	31	***Ifit1***	**20.3**	**2.5**	**8.1**	*0*.*25*	**2.23**
	50	***Ifi202b***	**6.1**	1.2	**5.0**	0.69	1.48
	69	***Zc3h12c***	**3.1**	**3.8**	0.8	1.65	1.78
Fig 4(astrocytes)	7	***Elovl7***	1.9	**2.9**	0.7	0.97	1.52
	8	***Cxcl11***	**95.4**	**10.6**	**9.0**	0.65	1.90
	4	***BC023105***	**78.0**	**3.4**	**23.1**	*0*.*41*	1.95
	3	***Rapgef5***	1.4	**2.6**	0.5	0.62	1.17

a: bold cells: increase >2 fold, italicize cells: decrease > 2 fold b: fold change in gene expression from CpG-ODN stimulated cells compared to mock as determined by real-time PCRd: difference between microglia and astrocytes in gene expressione: difference in fold expression induced by imiquimod vs CpG-ODN stimulation as determine by real-time PCR

### RNA Isolation and Quantitative Real-Time RT-PCR

At specified time-points post stimulation, astrocyte and microglia cells were lysed and processed for RNA extraction using a Mini RNA isolation kit (Zymo Research) following the manufacturer’s instructions. RNA was treated with DNAse I (Ambion) for 30 min at 37°C to remove any genomic DNA contamination and purified using RNA cleanup columns (Zymo Research).

cDNA was generated using an iScript reverse transcription kit (Bio-Rad) following the manufacturer’s instructions and included DNA contamination controls that did not undergo reverse transcription. cDNA samples were diluted fivefold in RNase-free water after reverse transcription, prior to use in quantitative real-time PCR reaction. All the real-time PCR reactions were completed using a Viia7 instrument (Applied Biosystems). All the samples were run in triplicate on a 384-well plate. Each reaction contained iTaq SYBR green supermix with ROX (Bio-Rad), 0.5 μM forward and reverse primers, approximately 10 ng of cDNA template and nuclease-free water. Primers were confirmed to be specific for the gene of interest. No homology to other genes was detected by basic local alignment search tool (BLAST) analysis of primers against the National Center for Biotechnology Information (NCBI) database. Dissociation curves were used to confirm amplification of a single product for each primer pair per sample. Data were calculated as the difference in C_T_ value (log2) for *Gapdh* mRNA minus the C_T_ value of the gene of interest for each sample (ΔCT = CT *Gapdh* −CT gene of interest) to control for variations in RNA amounts in each sample. Data was then calculated as fold change for each gene of interest relative to gene expression in mock-infected controls for each cell type.

## Results

### Transcriptome comparison between microglia and astrocytes following TLR7 activation

To directly compare how innate immune activation altered the gene expression profile in astrocytes and microglia, we used primary cultures of both cells types generated from neonatal mice. For TLR7 stimulation, we used 5 uM imiquimod for stimulation as this has previously shown TLR7-specific stimulation in both cell types [20]. Six hours was chosen as the optimal time point for analysis based on previous PCR array data that showed optimal induction of immune response genes at this time point post stimulation [20].

**Fig 1:**
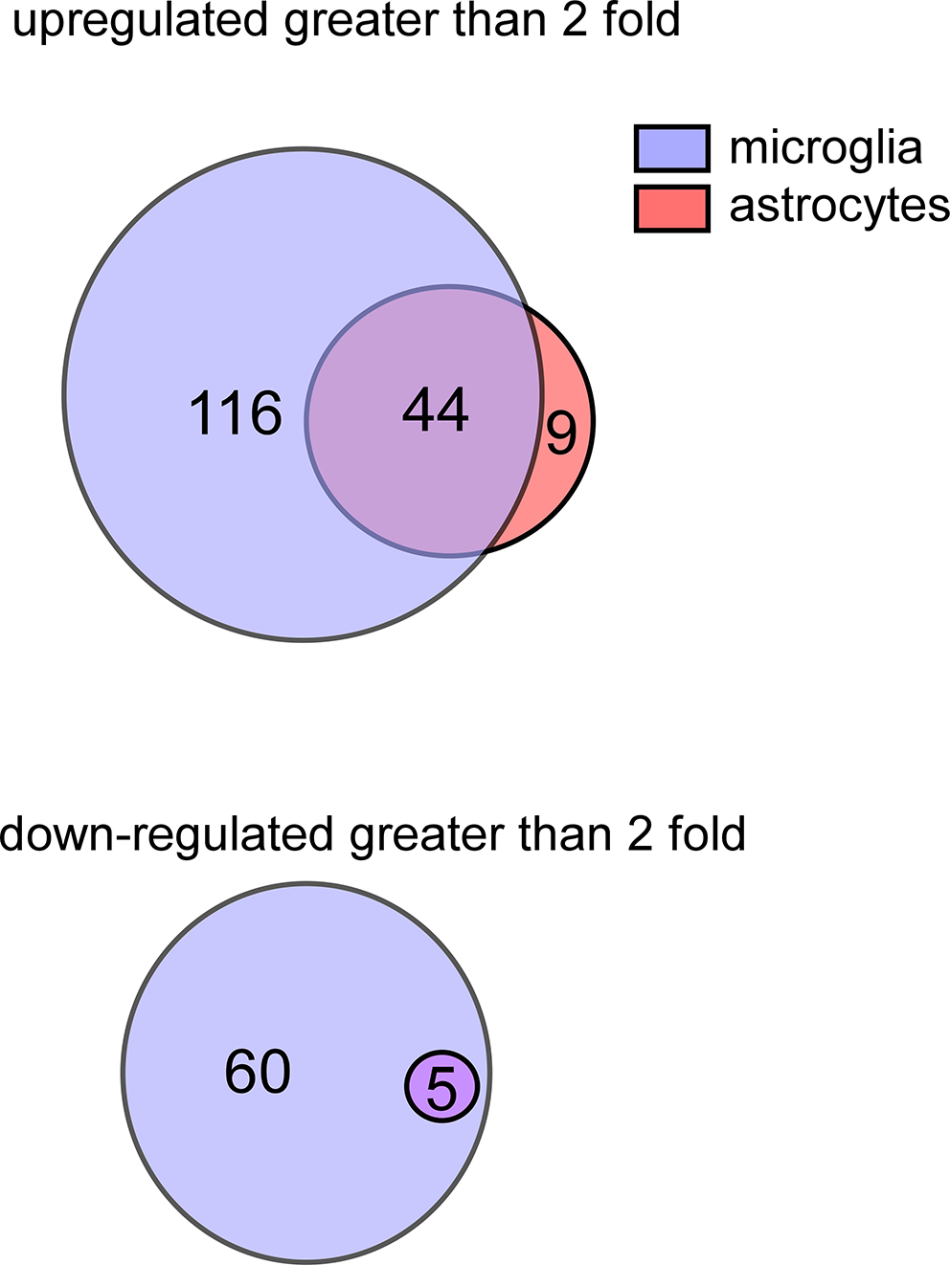
Venn diagram of genes with altered expression in microglia and astrocytes by TLR7 stimulation. The number of genes significantly (A) induced or (B) down-regulated at 6 hours post stimulation (hps) in microglia (blue circles) or astrocytes (red circle) as determined by microarray analysis, with genes expressed by both cell types displayed as overlapping circles. Microglia had the largest number of genes induced or down-regulated by TLR7 stimulation, with a total of 160 genes upregulated and 60 genes down-regulated. In contrast, only 53 genes were induced in astrocytes and 5 genes down-regulated. All down-regulated genes in astrocytes were also down-regulated in microglia, while 44 of the upregulated genes in astrocytes were also upregulated in microglia. All genes identified as upregulated or down-regulated were changed at least 2-fold compared to mock controls for each cell type and were found to be significantly altered (P <0.05) compared to mock-infected controls. Data is the average of 6 mock and 6 stimulated samples for each cell type.

**Fig 2:**
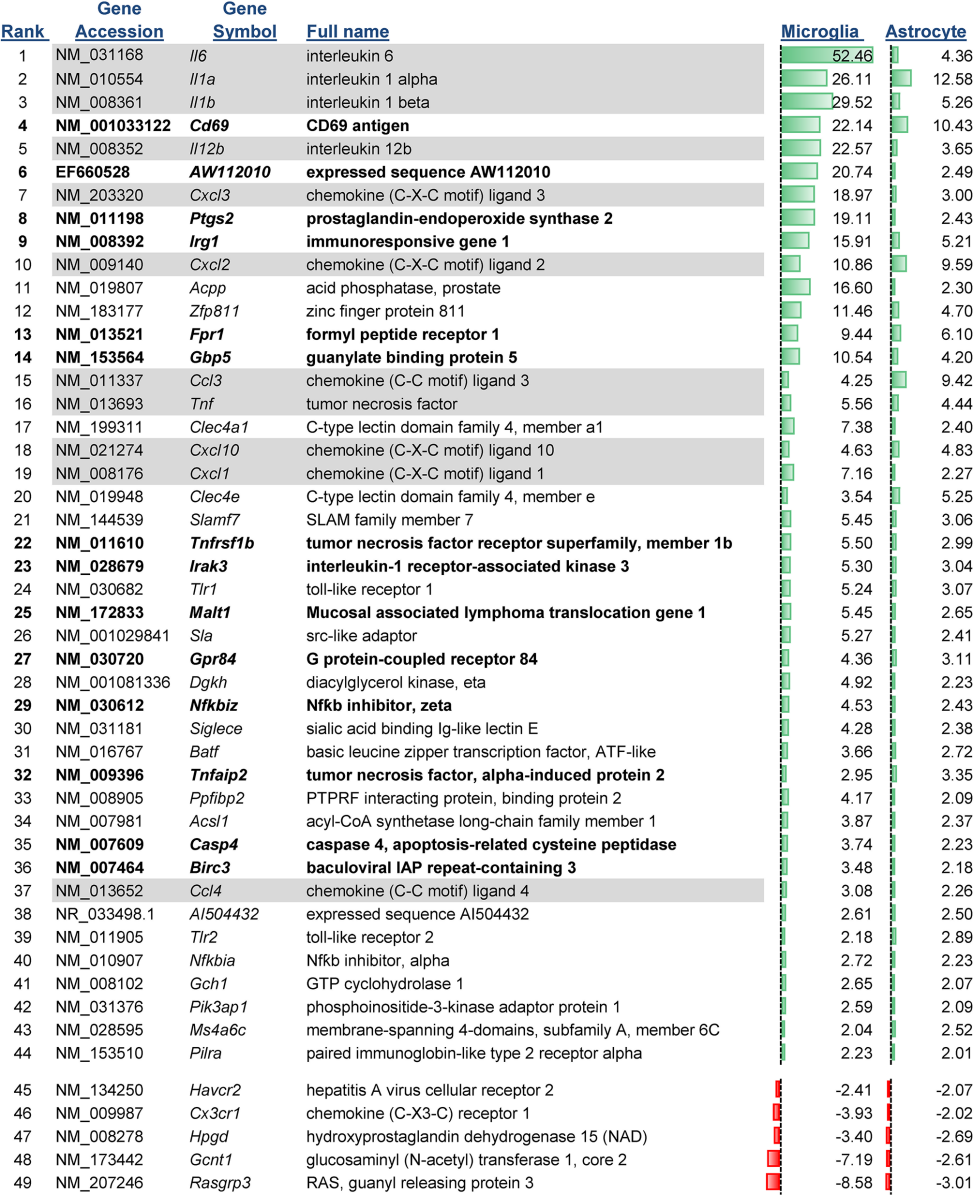
Ranking of Genes with expression change in both microglia and astrocytes. Genes with mRNA that were upregulated or down-regulated in both microglia and astrocytes by at least two-fold were graphed according the average increase between both microglia and astrocytes. Data are the mean fold increase relative to mock-infected samples for each cell type. n = 6 for each group including mock groups. Green bars indicate the relative increase and red bars indicate the relative decrease compared to mock over the range of all upregulated genes with a high value of 52.6 (interleukin 6) and a low value of -8.58 (Rasgrp3). Gene that are cytokines or chemokines are shaded, while genes that were chosen for further analysis by real-time PCR are shown in bold. The rank number is shown on the left side of the gene name.

Analysis of microarray data indicated a total of 293 genes with altered mRNA expression in microglia and/or astrocytes following TLR7 stimulation, with 169 genes significantly upregulated by at least two-fold and 60 genes that were down-regulated (Fig 1). Surprisingly, only 49 of the 293 genes were common to both microglia and astrocytes (Figs 1–2). Of the common upregulated genes, 10 of the top 20 were proinflammatory cytokines (Fig 2, shaded gray genes) correlating with the strong cytokine production associated with both cell types following TLR activation [20]. Only five genes had mRNA expression down-regulated in both astrocytes and microglia following TLR7 stimulation, including *Rasgrp3*, whose protein functions to inhibit TLR responses in macrophages [34].

### Microglia-specific responses to TLR7 stimulation by microarray analysis

**Fig 3:**
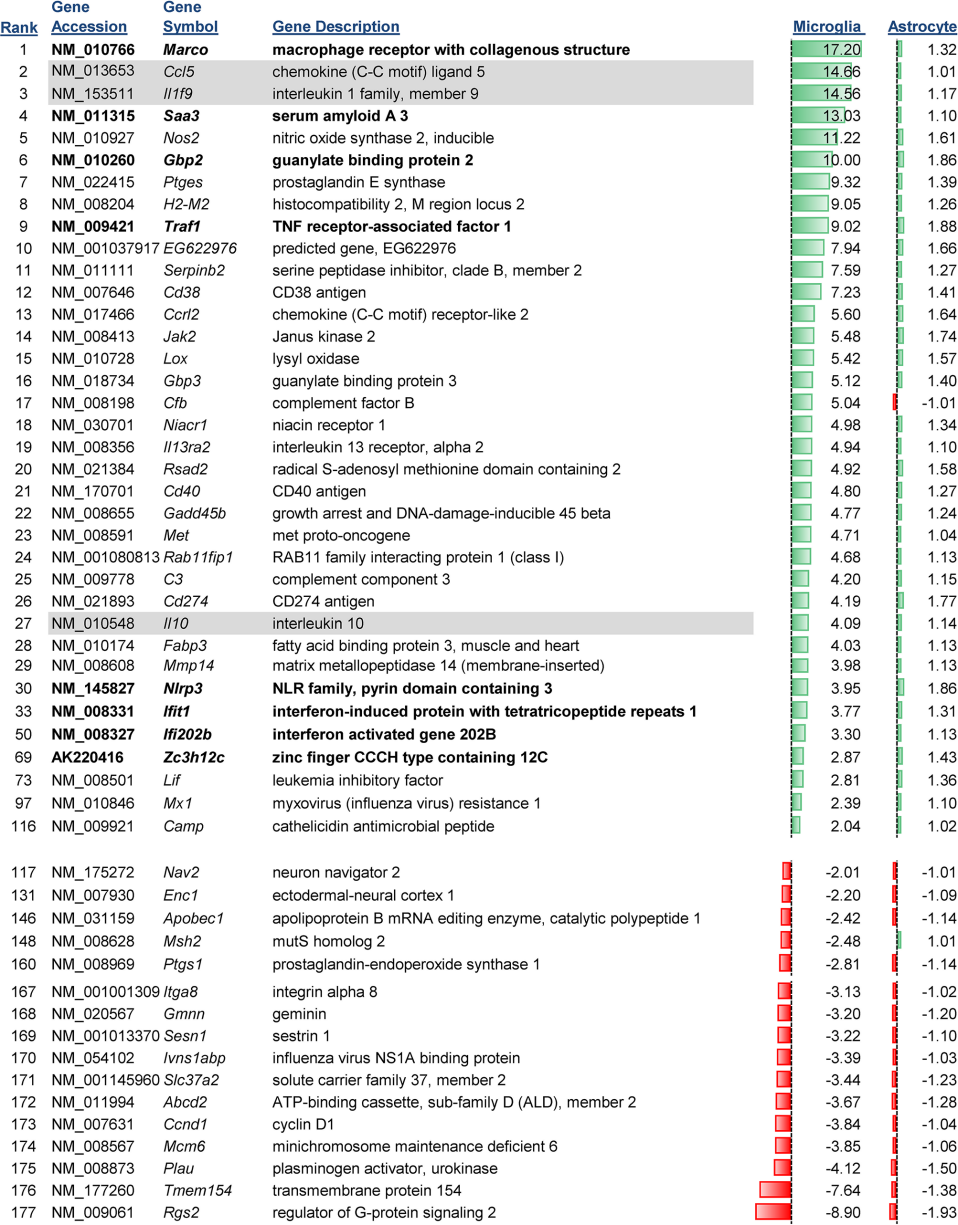
Genes with expression change only in microglia and not astrocytes. Genes whose mRNA expression was upregulated or down-regulated in microglia but not astrocytes were graphed according to their average fold increase in microglia. This data is only a partial list of genes and the full set of genes is shown in S1 Fig. Data for astrocytes are also shown. Data are the mean fold increase relative to mock-infected samples for each cell type. n = 6 for each group including mock groups. Green bars indicate the relative increase over the range of all upregulated genes with a high value of 17.2 (Marco) and a low value of -8.90 (Rgs2). Gene that are cytokines or chemokines are shaded, while genes that were chosen for further analysis by real-time PCR are shown in bold. The rank number is shown on the left side of the gene name.

Most of the alterations in gene expression were observed in microglia, with mRNA expression for 116 genes upregulated and 60 genes that were down-regulated (Fig 3 selected genes, full list in S1 Fig). Of the genes with upregulated mRNA expression, 28 were increased at least 4 fold indicating that TLR7 had a substantial impact on the transcriptome of microglia. Several of the genes with increased mRNA expression are well known activation markers of microglia including *Marco* and *Nos2* [35, 36]. However, other genes including *Saa3*, which to our knowledge had not been previously associated with microglia activation, were also upregulated and may provide additional markers for microglia responses in the CNS. Analysis of the changes in gene expression following TLR7 of microglia by IPA identified pathways linked to the activation of immune cells with the highest Z scores, followed by pathways associated with the recruitment and migration of cells (Table 1). These results are consistent with the role of microglia as the “immune cell of the CNS” and the ability of microglia to respond to sites of insult or injury in the CNS.

### Transcriptome response of astrocytes to TLR7 stimulation is less robust than microglia response

**Fig 4:**
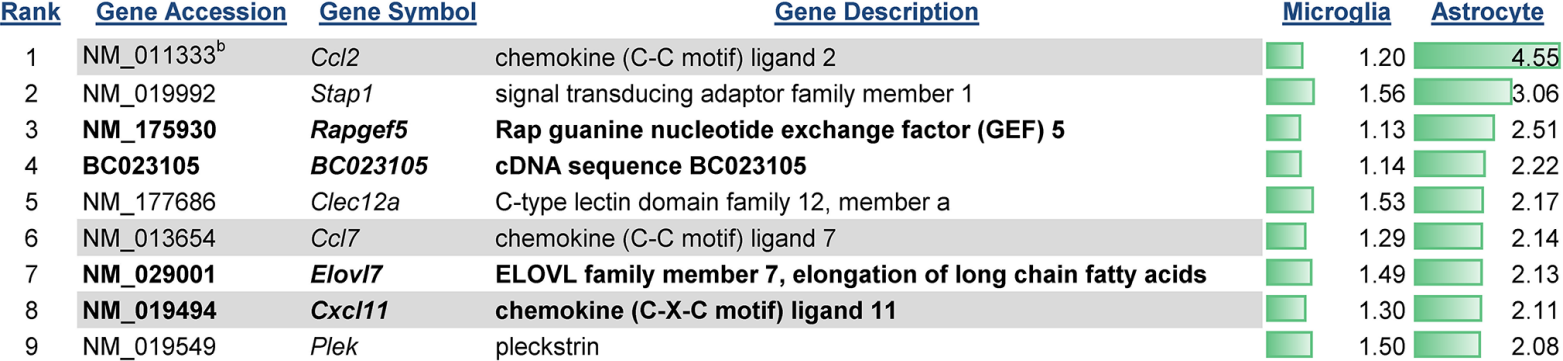
Genes with expression change in astrocytes and not microglia. Genes whose mRNA expression was upregulated or down-regulated in astrocytes but not microglia were graphed according to their average fold increase in astrocytes. Data are the mean fold increase relative to mock-infected samples for each cell type. n = 6 for each group including mock groups. Green bars indicate the relative increase over the range of all upregulated genes with a high value of 4.55 (*Ccl2*) and low value of 2.08 (*Plek*). Gene that are cytokines or chemokines are shaded, while genes that were chosen for further analysis by real-time PCR are shown in bold. The rank number is shown on the left side of the gene name.

TLR7 stimulation of astrocytes resulted in altered expression of a much smaller subset of genes as detected by microarray analysis, with only 53 genes upregulated by two-fold or greater, and five down-regulated. Of these, only 9 genes were specific to astrocyte activation (Figs 1 and 4). The only gene specific to astrocytes with greater than four-fold upregulation was *Ccl2*, a chemokine known to be highly expressed by astrocytes following infection or injury in the CNS [37, 38]. TLR7-induced changes in astrocyte transcriptome were found to be most similar to that of activated immune cells as determined by IPA analysis. This finding is similar to microglia and demonstrates innate immune activation of both cell types.

To determine if the activation of specific pathways differed between microglia and astrocytes, we calculated the greatest differences in IPA pathway Z scores. Pathways scoring higher with microglia relative to astrocytes were associated with lymphoproliferative diseases (Table 1) and correlated with the down regulation of cyclin D (*Ccnd1*) and the DNA mismatch repair gene MutS homolog 2 (*Msh2*) mRNA (Figs 3 and 4). The pathways that scored higher with astrocytes relative to microglia were associated with quantity of immune responses or cell cycle progression (Table 1). Thus, the biggest difference between microglia and astrocyte responses by IPA analysis was the difference in cell proliferation observed with microglia. This may reflect the proliferation of microglia following immune activation.

### Real-time PCR analysis of gene expression changes in TLR7-activated astrocytes increase sensitivity of detection

The production of proinflammatory cytokines and chemokines such as IL6, IL1α, IL1β, CCL2, CCL3, CCL4 and CCL5 has previously been associated with glial activation [20, 22, 39]. However, this study identified several genes besides these cytokines that are differentially induced in microglia and astrocytes. To better examine the expression of these genes between microglia and astrocytes, we utilized real-time PCR analysis, which is more sensitive in detecting transcript expression relative to microarray analysis [40]. We focused on genes that were induced by TLR activation in a) both cell types, b) microglia alone or c) astrocytes alone and that were upregulated at least two-fold (Table 2). Real-time PCR detection of gene expression was more sensitive than that observed with microarray when calculated as fold change relative to mock stimulated controls. Interestingly, TLR-induced gene expression was more readily detected in both cell populations for all but three of the selected genes (Table 2, second column). Only *Ifit1* was not induced by TLR-stimulation of astrocytes, whereas only *Elovl7* and *Rapgef5* were not upregulated by microglia (Table 2).

### Gene expression pattern similar following stimulation of another endosomal receptor, TLR9

**Fig 5:**
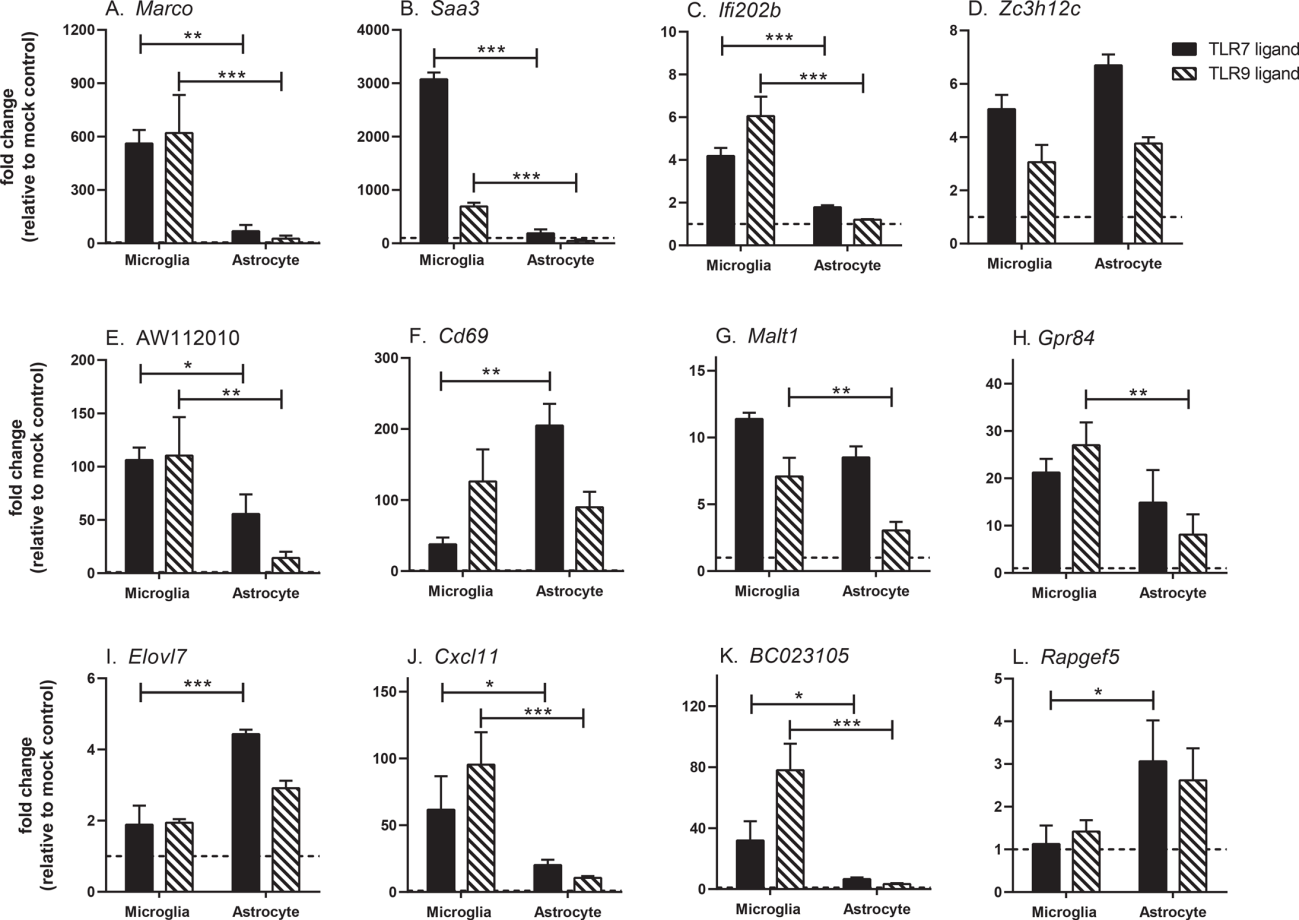
Real-time PCR analysis of mRNA expression in microglia and/or astrocytes following TLR7 or TLR9 stimulation. RNA samples from mock, TLR7 or TLR9-stimulated microglia or astrocytes were analyzed at 6 hps for mRNA expression of genes that were found by microarray to be (A-D) induced in microglia only, (E-H) induced in both microglia and astrocytes, or (I-L) induced in astrocytes only. Data are shown as the fold induction relative to mock-stimulated controls for each cell type (average of 6 mock-stimulated samples). Data are the mean +/- SEM for 3–6 samples per group and are representative of two separate experiments. Statistical analysis was conducted using a one-way ANOVA to determine differences between microglia and astrocytes for each stimulation. * P<0.05, ** P<0.01, *** P<0.001.

To determine if the above gene expression changes were a common response to TLR-induced activation of microglia and/or astrocytes, we stimulated both cell types with another ligand for an endosomal TLR, umethylated CpG-rich-oligodeoxynucleotides (CpG-ODN). Unmethylated CpG-rich DNA is produced by bacterial and some viral pathogens of the CNS and stimulates cells through TLR9, an endosomal TLR similar to TLR7. TLR9-induced activation of microglia and astrocytes induced a very similar response to TLR7-induced activation (Fig 5, Table 3). The main differences between TLR7 and TLR9 stimulation was higher mRNA upregulation of *Saa3* and *Traf1* by TLR7 stimulation and higher mRNA upregulation of *Ifit1*, *Nlrp3*, *Cd69*, BC023105 by TLR9 stimulation in microglia (Fig 5, Table 3). Thus, TLR9-induced activation of microglia and astrocytes induced similar genes to that observed with TLR7-induced activation, although the fold induction varied between the two stimuli.

### Induction of gene expression by viral infection in the CNS

**Fig 6:**
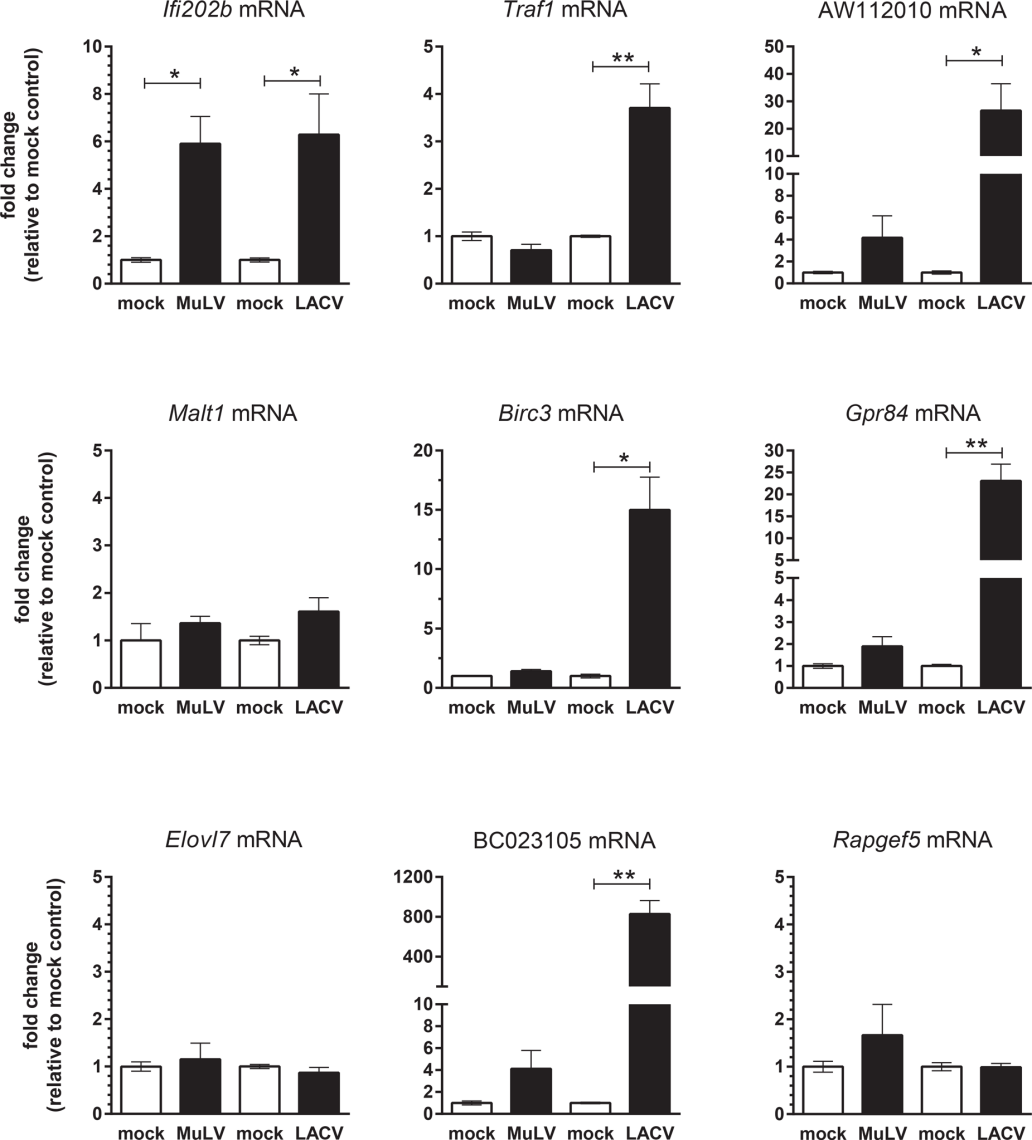
Real-time PCR analysis of mRNA expression of selected genes in brain tissue of mice with viral encephalitis. Brain tissue from mice with signs of neurological disease following infection with MuLV or LACV was processed for RNA. Age-matched and strain-matched controls for each virus infection were processed at the same time as viral infection and are shown as controls for the respective viruses. RNA was then analyzed for expression of mRNAs of genes identified as being induced following TLR activation of microglia and/or astrocytes. Data are the mean +/- SEM of 3–6 mice per group and are shown as the fold change relative to the average mock sample for each group. Statistical analysis was completed by unpaired t test between the virus-infected brain tissue and the appropriate mock-infected control. P<0.05, ** P<0.01, *** P<0.001.

The above studies indicate that several genes may be useful markers of microglia and astrocyte activation *in vitro*. We next determined if any of these identified genes were induced *in vivo* following virus infection of the CNS. We utilized two different mouse models of virus infection: a retrovirus (MuLV) infection model, where the primary cells infected are microglia and macrophages and the level of CNS inflammation is minimal, and La Crosse virus (LACV) infection, which results in substantial neuronal cell death and inflammatory infiltrate in the CNS. In both model systems, gliosis is associated with pathogenesis [41, 42]. We analyzed nine genes that were upregulated by TLR stimulation of either microglia and/or astrocytes in both systems. Interestingly, the two genes whose mRNAs were consistently only upregulated in astrocytes, *Elovl7* and *Rapgef5*, were not elevated in brain tissue following either virus infection (Fig 6). Only *Ifi202b* mRNA was significantly induced in brain tissue from MuLV-infected mice, which correlated with the lower level of inflammation in this model (Fig 6). However, several additional genes including *Traf1*, AW11202010, *Birc3*, *Gpr84* and BC023105 were upregulated following LACV infection. The upregulation of these genes by TLR stimulation of glial cells in vitro and virus infection in vivo suggests that mRNA expression of these genes may be useful for measuring glial activation in the CNS during virus infections.

## Discussion

In the current study, we defined the changes in the transcriptome of microglia and astrocytes in response innate immune activation via TLR7 or TLR9 stimulation. Although both astrocytes and microglia have previously been shown to respond to TLR activation through cytokine production [14, 20, 22, 39], the current study provides a more in-depth view of the response of both microglia and astrocytes to endosomal TLR activation. Microglia were found to induce a much broader response than astrocytes following TLR7 activation (Figs 1–4). However, some of this difference is likely to be due to the lower basal mRNA expression of many of these genes in astrocytes. Additionally, TLR7 and TLR9 are expressed at higher levels in microglia than in astrocytes [20], which could account for more stimulation in microglial cells and thus the higher overall number of genes induced by TLR stimulation.

The difference in detection of upregulated genes by real-time PCR in both astrocytes and microglia is most likely due to the amplification steps in PCR that allow detection of differences between lower copy numbers of mRNA transcripts that might not be detected by direct binding studies with microarray analysis [40]. Importantly, all of the genes found to be upregulated in a specific cell type by microarray were also upregulated by real-time PCR (Table 2), although not necessarily with the same fold increase between the two cell types. This indicates that although the level of detection and fold-increase may vary between the two methods, the genes that were detected as upregulated by microarray analysis could be confirmed by real-time PCR analysis. The fold change in cytokine mRNA expression is also highly dependent on the initial transcript level, particularly in the case of real-time PCR. Therefore, the relative fold-change per cell type may vary significantly between detection by real-time PCR versus that observed with microarray analysis [40]. This may also explain why the relative fold-change in cytokine mRNA expression is not consistent with protein expression. The microarray and real-time PCR analysis of cytokine genes in the current study indicate substantial differences in cytokine expression between microglia and astrocytes following TLR7 stimulation, including *Il-6*, *Il1a* and *Il-12*. However, protein analysis of these same cytokines showed similar levels in culture supernatants between astrocytes and microglia following TLR activation [20]. Thus, fold increase in gene expression is an indication of the upregulation of the mRNA, but is not a true representation of overall protein production.

The current microarray and real-time PCR analysis indentified several genes that may be important in CNS immune responses and distinguishing microglia and astrocyte responses. For example, *Ifi202b*, an interferon stimulatory gene (ISG) that is most known for its role as a lupus susceptibility gene in mice [43], was consistently only induced in microglia and not in astrocyte as measured by both microarray and real-time PCR (Fig 3, Table 2). Thus, *Ifi202b* may be a unique transcript that is upregulated primarily by microglia in response to TLR activation. *Ifi202b* mRNA was induced in the brain following MuLV and LACV infection (Fig 6) and was recently found to be induced in the CNS following Japanese encephalitis virus (JEV) infection [44]. IFI202b is a transcriptional regulator which has been shown to down-regulate AIM2 inflammasome signaling and regulate interferon (IFN) stimulated gene (ISG) expression [45]. Interestingly, the *Ifi202b* gene is truncated by a microdeletion in the 5’ flanking region and first exon in C57BL/6 mice, which affects the transcriptional expression in most, but not all, tissues [46]. Our current study indicates that *Ifi202b* mRNA is expressed in brain tissue from LACV-infected C57BL/6 mice (Fig 6). Thus, *Ifi202b* may be differentially regulated in the brain relative to other tissues.

In addition to differences between microglia and astrocytes, the microarray analysis detected genes that were differentially induced between TLR7 and TLR9 stimulation. For example, *Saa3* mRNA was upregulated at much higher levels in microglia following TLR7 stimulation compared to TLR9 stimulation (Fig 5). SAA3 protein is involved in the activation of the NLRP3 inflammasome [47]. The NLRP3 inflammasome cleaves pro-IL1β to IL-1β, which can mediate cellular damage in the brain by causing pyroptosis [48]. SAA3 can prime glial cells to produce IL-1 at comparable levels to that observed by LPS stimulation [49]. Thus, the substantially higher increase in *Saa3* mRNA in microglia following TLR7 stimulation, as compared to TLR9 stimulation, may result in higher levels of active IL-1β, which could have a substantial effect on the inflammatory response in the CNS.

Several other transcripts, including *Traf1*, *Birc3* and *Gpr84*, were upregulated both *in vitro* (Fig 5) and *in vivo* (Fig 6) and may be useful for analysis of microglia and/or astrocyte activation. *Traf1* and *Birc3* products are members of the NFκB canonical and non-canonical signaling pathways that have recently been shown to interact with each other to regulate TNF signaling [50]. Increased transcription of these two genes may be essential for microglia and/or astrocytes to respond to subsequent immune stimuli. Another upregulated transcript, *Gpr84*, produces a protein that is expressed primarily on myeloid cells, recognizes medium chain fatty acids and is involved in immune regulation. Interestingly, a two base pair frameshift deletion in *Gpr84* has been identified in several mouse strains including DBA/1, FVB/NJ, NOD and SJL/J [51]. Since *Gpr84* is induced in both microglia and astrocytes, this frameshift deletion could affect the responses of glial cells to infection in these mouse strains.

This study also identified two expressed sequences, AW112010 and BC023105, whose transcripts were upregulated in both microglia and astrocytes following TLR stimulation and were induced in the CNS following LACV infection. BC023105 is a pseudogene, and therefore may be a marker of inflammation without influencing glial cells responses [52]. AW112010 is a protein coding gene, whose function has not yet been identified. However, the consistent expression of AW112010 RNA by immune activation suggests that AW112010 may encode a protein involved in immune function, which may warrant further analysis.

## Conclusions

In the current study, we compared the transcriptome changes of microglia and astrocytes following stimulation of TLR7, an endosomal TLR that recognizes both PAMPs and DAMPs in the CNS. Microarray analysis demonstrated that TLR stimulation induces the upregulation and down-regulation of mRNAs for substantially more genes in microglia than in astrocytes. However, one explanation for this difference may be due to the sensitivity of microarray analysis, since real-time PCR detected transcriptional changes of the same genes in astrocytes, although not to the same fold-increase as microglia. Analysis of a subset of these genes identified at least one gene, *Ifi202b*, which was specific to microglia and was upregulated not only in vitro, but also induced by virus infection, *in vivo*. Other genes, including *Gpr84* and *Birc3*, as well as expressed transcripts AW112010 and BC023105 were consistently upregulated in activated microglia and astrocytes and were also upregulated in the CNS following virus infection. Thus, these genes may be useful markers of glial activation following infection or damage in the CNS.

## Supporting Information

S1 Fig Full list of genes upregulated only in microglia and not astrocytes. Genes whose mRNA expression was upregulated or down-regulated in microglia but not astrocytes were graphed according to their average fold increase in microglia. Data for astrocytes are also shown. Data are the mean fold increase relative to mock-infected samples for each cell type. n = 6 for each group including mock groups. Green bars indicate the relative increase over the range of all upregulated genes with a high value of 17.2 (Marco) and a low value of -8.90 (Rgs2).
(PDF)
