# Supplementary material for: Identification of Glial Activation Markers by Comparison of Transcriptome Changes between Astrocytes and Microglia following Innate Immune Stimulation
Source: PLoS One. 2015 Jul 27;10(7):e0127336. doi: 10.1371/journal.pone.0127336 (PMC4516330; doi:10.1371/journal.pone.0127336)
Supplement: S1 Fig — Genes whose mRNA expression was upregulated or down-regulated in microglia but not astrocytes were graphed according to their average fold increase in microglia. Data for astrocytes are also shown. Data are the mean fold increase relative to mock-infected samples for each cell type. n = 6 for each group including mock groups. Green bars indicate the relative increase over the range of all upregulated genes with a high value of 17.2 (Marco) and a low value of -8.90 (Rgs2). (PDF) [file pone.0127336.s001.pdf]

S1 Fig. Genes upregulated only in microglia and not astrocytes. Genes whose mRNA expression was upregulated or down-regulated in microglia but not astrocytes were graphed according to their average fold increase in microglia. All genes are presented (partial graph of genes is shown in Fig. 3). Data for astrocytes are also shown. Data are the mean fold increase relative to mock-infected samples for each cell type. n = 6 for each group including mock groups. Green bars indicate the relative increase over the range of all upregulated genes with a high value of 17.2 (Marco) and a low value of -8.90 (Rgs2).

Rank	Gene Accession	Gene Symbol	Gene Description	Microglia	Astrocyte
1	NM_010766	*Marco*	macrophage receptor with collagenous structure	17.20	1.32
2	NM_013653	*Ccl5*	chemokine (C-C motif) ligand 5	14.66	1.01
3	NM_153511	*Il1f9*	interleukin 1 family, member 9	14.56	1.17
4	NM_011315	*Saa3*	serum amyloid A 3	13.03	1.10
5	NM_010927	*Nos2*	nitric oxide synthase 2, inducible	11.22	1.61
6	NM_010260	*Gbp2*	guanylate binding protein 2	10.00	1.86
7	NM_022415	*Ptges*	prostaglandin E synthase	9.32	1.39
8	NM_008204	*H2-M2*	histocompatibility 2, M region locus 2	9.05	1.26
9	NM_009421	*Traf1*	TNF receptor-associated factor 1	9.02	1.88
10	NM_001037917	*EG622976*	predicted gene, EG622976	7.94	1.66
11	NM_011111	*Serpinb2*	serine (or cysteine) peptidase inhibitor, clade B, member 2	7.59	1.27
12	NM_007646	*Cd38*	CD38 antigen	7.23	1.41
13	NM_017466	*Ccrl2*	chemokine (C-C motif) receptor-like 2	5.60	1.64
14	NM_008413	*Jak2*	Janus kinase 2	5.48	1.74
15	NM_010728	*Lox*	lysyl oxidase	5.42	1.57
16	NM_018734	*Gbp3*	guanylate binding protein 3	5.12	1.40
17	NM_008198	*Cfb*	complement factor B	5.04	-1.01
18	NM_030701	*Niacr1*	niacin receptor 1	4.98	1.34
19	NM_008356	*Il13ra2*	interleukin 13 receptor, alpha 2	4.94	1.10
20	NM_021384	*Rsad2*	radical S-adenosyl methionine domain containing 2	4.92	1.58
21	NM_170701	*Cd40*	CD40 antigen	4.80	1.27
22	NM_008655	*Gadd45b*	growth arrest and DNA-damage-inducible 45 beta	4.77	1.24
23	NM_008591	*Met*	met proto-oncogene	4.71	1.04
24	NM_001080813	*Rab11fip1*	RAB11 family interacting protein 1 (class I)	4.68	1.13
25	NM_009778	*C3*	complement component 3	4.20	1.15
26	NM_021893	*Cd274*	CD274 antigen	4.19	1.77
27	NM_010548	*Il10*	interleukin 10	4.09	1.14
28	NM_010174	*Fabp3*	fatty acid binding protein 3, muscle and heart	4.03	1.13
29	NM_008608	*Mmp14*	matrix metallopeptidase 14 (membrane-inserted)	3.98	1.13
30	NM_145827	*Nlrp3*	NLR family, pyrin domain containing 3	3.95	1.86
31	NM_017370	*Hp*	haptoglobin	3.94	1.31
32	NM_010104	*Edn1*	endothelin 1	3.86	1.20
33	NM_008331	*Ifit1*	interferon-induced protein with tetratricopeptide repeats 1	3.77	1.31
34	NM_009137	*Ccl22*	chemokine (C-C motif) ligand 22	3.76	1.23
35	NM_010407	*Hck*	hemopoietic cell kinase	3.75	1.41
36	NM_008360	*Il18*	interleukin 18	3.67	1.25
37	NM_025967	*D16Ertd472e*	DNA segment, Chr 16, ERATO Doi 472, expressed	3.65	1.23
38	NM_009647	*Ak3l1*	adenylate kinase 3-like 1	3.64	1.16
39	NM_033322	*Lztfl1*	leucine zipper transcription factor-like 1	3.60	1.12
40	NM_028935	*Zfp558*	zinc finger protein 558	3.58	-1.04
41	NM_194336	*Mpa2l*	macrophage activation 2 like	3.56	1.74
42	NM_007707	*Socs3*	suppressor of cytokine signaling 3	3.55	1.23
43	NM_011990	*Slc7a11*	solute carrier family 7, member 11	3.51	1.65
44	NM_023908	*Slco3a1*	solute carrier organic anion transporter family, member 3a1	3.49	1.18
45	NM_010501	*Ifit3*	interferon-induced protein with tetratricopeptide repeats 3	3.49	1.00
46	NM_008332	*Ifit2*	interferon-induced protein with tetratricopeptide repeats 2	3.46	1.05
47	NM_008607	*Mmp13*	matrix metallopeptidase 13	3.39	1.24
48	NM_028523	*Dcbld2*	discoidin, CUB and LCCL domain containing 2	3.32	1.18
49	NM_013642	*Dusp1*	dual specificity phosphatase 1	3.31	-1.03
50	NM_008327	*Ifi202b*	interferon activated gene 202B	3.30	1.13
51	NM_133232	*Pfkfb3*	6-phosphofructo-2-kinase/fructose-2,6-biphosphatase 3	3.27	1.06
52	NM_013831	*Pstpip2*	proline-serine-threonine phosphatase-interacting protein 2	3.16	1.47
53	NM_019840	*Pde4b*	phosphodiesterase 4B, cAMP specific	3.13	1.52

54	BC049633	*AA467197*	expressed sequence AA467197	3.10	1.52
55	NM_010274	*Gpd2*	glycerol phosphate dehydrogenase 2, mitochondrial	3.07	-1.04
56	NM_001033302	*Gm129*	gene model 129, (NCBI)	3.07	-1.12
57	NM_009344	*Phlda1*	pleckstrin homology-like domain, family A, member 1	3.07	1.46
58	NM_172050	*Cd300e*	CD300e antigen	3.05	1.10
59	NM_009477	*Upp1*	uridine phosphorylase 1	3.03	1.20
60	NR_003508	*Mx2*	myxovirus (influenza virus) resistance 2	3.02	1.07
61	NM_015766	*Ebi3*	Epstein-Barr virus induced gene 3	2.99	1.30
62	NM_013599	*Mmp9*	matrix metallopeptidase 9	2.98	1.43
63	NM_133209	*Pilrb1*	paired immunoglobin-like type 2 receptor beta 1	2.95	1.20
64	NM_013790	*Abcc5*	ATP-binding cassette, sub-family C, member 5	2.95	1.13
65	NM_028133	*Egln3*	EGL nine homolog 3 (C. elegans)	2.90	1.05
66	NM_133661	*Slc6a12*	solute carrier family 6, member 12	2.90	1.07
67	NM_015783	*Isg15*	ISG15 ubiquitin-like modifier	2.88	1.07
68	NM_010809	*Mmp3*	matrix metallopeptidase 3	2.88	1.14
69	AK220416	*Zc3h12c*	zinc finger CCCH type containing 12C	2.87	1.43
70	NM_009627	*Adm*	adrenomedullin	2.86	1.17
71	NM_130447	*Dusp16*	dual specificity phosphatase 16	2.86	1.20
72	NM_008987	*Ptx3*	pentraxin related gene	2.82	1.19
73	NM_008501	*Lif*	leukemia inhibitory factor	2.81	1.36
74	NM_033322	*Lztfl1*	leucine zipper transcription factor-like 1	2.78	1.20
75	NM_001081211	*Ptafr*	platelet-activating factor receptor	2.77	-1.04
76	NM_001040400	*Tet2*	tet oncogene family member 2	2.75	1.02
77	NM_030259	*Rilpl2*	Rab interacting lysosomal protein-like 2	2.75	1.31
78	NM_009760	*Bnip3*	BCL2/adenovirus E1B interacting protein 3	2.74	1.14
79	NM_007413	*Adora2b*	adenosine A2b receptor	2.73	1.25
80	NM_001099624	*Rapgef2*	Rap guanine nucleotide exchange factor (GEF) 2	2.68	1.02
81	NM_008380	*Inhba*	inhibin beta-A	2.67	1.15
82	NM_011580	*Thbs1*	thrombospondin 1	2.67	-1.06
83	NM_001163567	*Fam102b*	family with sequence similarity 102, member B	2.66	-1.06
84	NM_181545	*Slfn8*	schlafen 8	2.66	1.27
85	NM_175549	*Robo2*	roundabout homolog 2 (Drosophila)	2.65	1.01
86	NM_025681	*Lix1*	limb expression 1 homolog (chicken)	2.63	1.01
87	NM_007972	*F10*	coagulation factor X	2.58	1.02
88	NM_007936	*Epha4*	Eph receptor A4	2.53	-1.07
89	NM_008215	*Has1*	hyaluronan synthase1	2.52	-1.01
90	NM_028680	*Ift57*	intraflagellar transport 57 homolog (Chlamydomonas)	2.51	1.06
91	NM_009971	*Csf3*	colony stimulating factor 3 (granulocyte)	2.50	1.08
92	NM_031167	*Il1rn*	interleukin 1 receptor antagonist	2.49	1.16
93	NM_130877	*Peg10*	paternally expressed 10	2.49	-1.17
94	NM_001102404	*Acp5*	acid phosphatase 5, tartrate resistant	2.45	1.11
95	NM_138648	*Olr1*	oxidized low density lipoprotein (lectin-like) receptor 1	2.41	1.17
96	NM_021524	*Nampt*	nicotinamide phosphoribosyltransferase	2.40	1.06
97	NM_010846	*Mx1*	myxovirus (influenza virus) resistance 1	2.39	1.10
98	NM_134080	*Flnb*	filamin, beta	2.38	-1.01
99	NM_010162	*Ext1*	exostoses (multiple) 1	2.37	1.14
100	NM_007616	*Cav1*	caveolin 1, caveolae protein	2.37	-1.08
101	NM_029942	*Prelid2*	PRELI domain containing 2	2.34	1.12
102	NM_008329	*Ifi204*	interferon activated gene 204	2.34	1.15
103	NM_146085	*Apbb3*	amyloid beta precursor protein-binding, family B, member 3	2.34	1.01
104	NM_026772	*Cdc42ep2*	CDC42 effector protein (Rho GTPase binding) 2	2.33	1.00
105	NM_212444	*Gyk*	glycerol kinase	2.31	1.11
106	NM_030678	*Gys1*	glycogen synthase 1, muscle	2.28	1.08
107	NM_009642	*Agtrap*	angiotensin II, type I receptor-associated protein	2.27	1.06
108	NM_008247	*Ppap2a*	phosphatidic acid phosphatase type 2A	2.25	1.06
109	NM_007494	*Ass1*	argininosuccinate synthetase 1	2.24	1.10
110	NM_001081249	*Vcan*	versican	2.23	1.05
111	NM_011171	*Procr*	protein C receptor, endothelial	2.22	1.07
112	NM_144839	*Ube2e2*	ubiquitin-conjugating enzyme E2E 2 (UBC4/5 homolog, yeast)	2.18	-1.03
113	NM_001033922	*Treml4*	triggering receptor expressed on myeloid cells-like 4	2.10	1.01
114	NM_031997	*Tmem2*	transmembrane protein 2	2.07	1.00
115	NM_153402	*Eif2c3*	eukaryotic translation initiation factor 2C, 3	2.05	1.01

116	NM_009921	*Camp*	cathelicidin antimicrobial peptide	2.04	1.02
117	NM_175272	*Nav2*	neuron navigator 2	-2.01	-1.01
118	NM_011851	*Nt5e*	5' nucleotidase, ecto	-2.05	1.02
119	NM_001113460	*Tec*	tec protein tyrosine kinase	-2.08	-1.03
120	NM_023794	*Etv5*	ets variant gene 5	-2.09	-1.04
121	NM_021886	*Cenph*	centromere protein H	-2.09	-1.01
122	NM_153139	*Slc36a1*	solute carrier family 36, member 1	-2.09	-1.01
123	NM_001030307	*Dkc1*	dyskeratosis congenita 1, dyskerin homolog	-2.10	1.00
124	NM_010931	*Uhrf1*	ubiquitin-like, containing PHD and RING finger domains, 1	-2.12	-1.02
125	NM_134081	*Dnajc9*	DnaJ (Hsp40) homolog, subfamily C, member 9	-2.12	1.01
126	NM_146069	*Lrrc33*	leucine rich repeat containing 33	-2.16	-1.05
127	NM_008563	*Mcm3*	minichromosome maintenance deficient 3	-2.17	1.08
128	NM_010137	*Epas1*	endothelial PAS domain protein 1	-2.17	-1.06
129	NM_001025382	*EG574403*	predicted gene, EG574403	-2.17	-1.05
130	NM_001113518	*Arhgef7*	Rho guanine nucleotide exchange factor (GEF7)	-2.19	-1.06
131	NM_007930	*Enc1*	ectodermal-neural cortex 1	-2.20	-1.09
132	NM_175429	*Kctd12b*	potassium channel tetramerisation domain containing 12b	-2.21	-1.04
133	BC046640	*Tmem173*	transmembrane protein 173	-2.23	1.03
134	NM_011249	*Rbl1*	retinoblastoma-like 1 (p107)	-2.23	-1.10
135	NM_172814	*Lrp12*	low density lipoprotein-related protein 12	-2.24	-1.09
136	NM_028904	*Rmi1*	RMI1, RecQ mediated genome instability 1, homolog	-2.26	-1.10
137	NM_001110231	*Cugbp2*	CUG triplet repeat, RNA binding protein 2	-2.27	-1.13
138	NM_024290	*Tnfrsf23*	tumor necrosis factor receptor superfamily, member 23	-2.27	1.00
139	NM_007691	*Chek1*	checkpoint kinase 1 homolog	-2.28	-1.04
140	NM_146040	*Cdca7l*	cell division cycle associated 7 like	-2.28	1.04
141	NM_008317	*Hyal1*	hyaluronoglucosaminidase 1	-2.33	-1.04
142	NM_175645	*Xylt1*	xylosyltransferase 1	-2.34	-1.07
143	NM_008921	*Prim1*	DNA primase, p49 subunit	-2.34	-1.09
144	NM_021790	*Cenpk*	centromere protein K	-2.35	-1.11
145	NM_172598	*Wdhd1*	WD repeat and HMG-box DNA binding protein 1	-2.39	-1.05
146	NM_031159	*Apobec1*	apolipoprotein B mRNA editing enzyme, catalytic polypeptide 1	-2.42	-1.14
147	NM_176837	*Arhgap18*	Rho GTPase activating protein 18	-2.42	-1.13
148	NM_008628	*Msh2*	mutS homolog 2	-2.48	1.01
149	NM_016920	*Atp6v0a1*	ATPase, H+ transporting, lysosomal V0 subunit A1	-2.56	-1.05
150	NM_001080814	*Fat3*	FAT tumor suppressor homolog 3	-2.57	-1.20
151	BC027537	*Gins1*	GINS complex subunit 1 (Psf1 homolog)	-2.60	-1.09
152	NM_008533	*Cd180*	CD180 antigen	-2.60	-1.06
153	NM_008565	*Mcm4*	minichromosome maintenance deficient 4 homolog	-2.64	-1.06
154	NM_001146180	*Mtss1*	metastasis suppressor 1	-2.65	-1.23
155	NM_001142918	*Tcf7l2*	transcription factor 7-like 2, T-cell specific, HMG-box	-2.67	-1.14
156	NM_134083	*Rcbtb2*	RCC1 and BTB domain containing protein 2	-2.71	-1.17
157	NM_146073	*Zdhhc14*	zinc finger, DHHC domain containing 14	-2.72	-1.16
158	NM_008892	*Pola1*	polymerase (DNA directed), alpha 1	-2.75	1.01
159	NM_177184	*Vps13c*	vacuolar protein sorting 13C (yeast)	-2.80	-1.23
160	NM_008969	*Ptgs1*	prostaglandin-endoperoxide synthase 1	-2.81	-1.14
161	NM_007420	*Adrb2*	adrenergic receptor, beta 2	-2.82	-1.04
162	NM_019971	*Pdgfc*	platelet-derived growth factor, C polypeptide	-2.85	-1.28
163	NM_139232	*Fgd4*	FYVE, RhoGEF and PH domain containing 4	-2.86	-1.07
164	NM_178407	*Arap2*	ArfGAP with RhoGAP domain, ankyrin repeat and PH domain 2	-2.92	1.03
165	NM_133829	*Mfsd6*	major facilitator superfamily domain containing 6	-2.95	-1.10
166	NM_008234	*Hells*	helicase, lymphoid specific	-2.96	-1.03
167	NM_001001309	*Itga8*	integrin alpha 8	-3.13	-1.02
168	NM_020567	*Gmnn*	geminin	-3.20	-1.20
169	NM_001013370	*Sesn1*	sestrin 1	-3.22	-1.10
170	NM_054102	*Ivns1abp*	influenza virus NS1A binding protein	-3.39	-1.03
171	NM_001145960	*Slc37a2*	solute carrier family 37, member 2	-3.44	-1.23
172	NM_011994	*Abcd2*	ATP-binding cassette, sub-family D (ALD), member 2	-3.67	-1.28
173	NM_007631	*Ccnd1*	cyclin D1	-3.84	-1.04
174	NM_008567	*Mcm6*	minichromosome maintenance deficient 6	-3.85	-1.06
175	NM_008873	*Plau*	plasminogen activator, urokinase	-4.12	-1.50
176	NM_177260	*Tmem154*	transmembrane protein 154	-7.64	-1.38
177	NM_009061	*Rgs2*	regulator of G-protein signaling 2	-8.90	-1.93
